# Effect of orthodontic treatment on alveolar bone thickness in adults: a systematic review

**DOI:** 10.1590/2177-6709.24.4.034-045.oar

**Published:** 2019

**Authors:** Michelle Sendyk, Daniele Sigal Linhares, Claudio Mendes Pannuti, João Batista de Paiva, José Rino

**Affiliations:** 1 Universidade de São Paulo, Departamento de Ortodontia e Odontopediatria, Divisão de Ortodontia (São Paulo/SP, Brazil).; 2 Universidade de São Paulo, Departamento de Estomatologia, Divisão de Periodontia (São Paulo/SP, Brazil).

**Keywords:** Orthodontics, Alveolar bone loss, Periodontics, Tooth movement

## Abstract

**Objectives:**

This review aimed at evaluating changes in alveolar bone thickness after completion of orthodontic treatment.

**Methods:**

Only prospective clinical studies that reported bone thickness in adult patients undergoing non-surgical orthodontic treatment were considered eligible. MEDLINE, EMBASE and LILACS databases were searched for articles published up to July 2018.

**Results:**

A total of 12 studies met the selected criteria. Most of the studies showed that orthodontic treatment produces a reduction in bone thickness of incisors, mainly at the palatal side.

**Conclusion:**

On patients undergoing different orthodontic treatment techniques, there was a significant bone thickness reduction, mainly on the palatal side.

**Clinical relevance:**

These findings are relevant and have to be considered in diagnosis and planning of tooth movement, in order to prevent the occurrence of dehiscence and fenestration in alveolar bone.

## RESUMO

**Objetivo:**

o objetivo da presente revisão foi avaliar as mudanças na espessura óssea alveolar após o término do tratamento ortodôntico.

**Métodos:**

as bases de dados MEDLINE, EMBASE e LILACS foram consultadas para artigos publicados até julho de 2018, sendo considerados elegíveis somente estudos prospectivos clínicos que relataram a espessura óssea em adultos submetidos ao tratamento ortodôntico não cirúrgico.

**Resultados:**

no total, 12 estudos atenderam aos critérios de inclusão, e a maioria dos estudos mostrou que o tratamento ortodôntico promove redução na espessura óssea dos incisivos, principalmente na face palatina.

**Conclusão:**

em pacientes submetidos a diferentes técnicas ortodônticas, foi observada uma redução significativa na espessura óssea, especialmente na face palatina.

**Relevância clínica:**

esses achados são relevantes e devem ser considerados no diagnóstico e plano de tratamento do movimento dentário, com o objetivo de prevenir a ocorrência de deiscências e fenestrações ósseas.

## INTRODUCTION

The longevity of a tooth depends on its periodontal health. Evidences show that orthodontic treatment can result in loss of periodontal support in the presence of plaque and inflammation.^1^ ^-^ ^3^ Orthodontic treatments that result in pronounced tooth inclinations are considered to be risk factors for dehiscence and fenestration. One possible factor related to these occurrences is the reduced thickness of the alveolar bone around the roots.^4^ Thus, it is important to treat with caution orthodontic patients who already have thin soft-tissue margins before treatment, since the buccal tooth movement may render the gingival tissue more vulnerable and less resistant to plaque and tooth brush trauma.^4^ ^-^ ^13^

The first attempt to delineate the effect of tooth movement on bone thicknesses concentrated on animal studies.^14^ ^,^ ^15^ Subsequently, human studies were conducted using lateral and frontal cephalometric radiographs.^16^ ^,^ ^17^ However, the radiographic methods are affected by the superimposition of anatomical structures, difficulties in identification of individual teeth and magnification errors.^18^

Currently it is possible to measure alveolar bone thickness around the roots using the images obtained by cone beam computed tomography (CBCT).^19^ ^-^ ^22^ The accuracy and reproducibility of CBCT are well documented in the literature.^23^ ^,^ ^24^ However, to our knowledge, no systematic review has evaluated the effects of orthodontic treatment on bone thickness using CBCT. 

Thus, the aim of the present systematic review (SR) is to evaluate the effects of orthodontic treatment on alveolar bone thickness, comparing different types of treatment techniques in adult patients. 

## MATERIAL AND METHODS

### Search strategy

The study protocol of this SR was registered at the National Institute for Health Research PROSPERO (International Prospective Register of Systematic Reviews, http://www.crd.york.ac.uk/prospero). The review text was structured in accordance with guidelines from PRISMA (Preferred Reporting Items for Systematic Reviews and Meta-Analyses) and the Cochrane Handbook of Systematic Reviews of Interventions.

Search strategies were developed for MEDLINE via PubMed, EMBASE and LILACS databases until July 2018. MesH terms and keywords were combined with Boolean operators and used to search the databases:

#1: (tomography OR cone beam computed tomography OR tridimensional OR CBCT OR cone-beam);
#2: (bone thickness OR alveolar thickness OR alveolar bone OR fenestration OR dehiscence OR width);
#3: (orthodontic OR malocclusion);

(#1 AND #2 AND #3). 

After the initial electronic search, the authors manually searched articles in the bibliographies of the included studies. 

### Inclusion and exclusion criteria 

Only randomized clinical trials, controlled clinical trials, case series and observational prospective studies with one or more orthodontic treatment arms and tridimensional evaluation of alveolar bone thickness before and after the orthodontic treatment in adult patients were considered eligible for inclusion in this review. Observational studies that included children, patients who had received orthopedic rapid maxillary expansion or accelerated orthodontic treatment such as perforation or corticotomies; studies in which bone thickness was not evaluated using CBCT and studies performed in patients with syndromes and cleft patients were excluded from the review. Further, animal studies, letter to the editors, reviews and *in vitro* studies were not included.

Different techniques of corrective orthodontic treatment were considered for this review, among them Straight-wire and Edgewise techniques, and extraction and non-extraction treatments.

The primary outcome was alveolar bone thickness change. The alveolar bone thickness was measured on maxillary or mandibular central and lateral incisors, upper canines, upper and lower premolars and evaluated at three different distances (3, 6 and 9 mm) from the cementoenamel junction (CEJ) (cervical, middle or apical).

### Data extraction

Data were extracted independently by two reviewers, and the disagreements were solved by discussion with a third reviewer. Studies appearing to meet the inclusion criteria or those with insufficient information in the title and abstract to allow a clear decision were selected for assessment of the full text, which was carried independently by the same two reviewers to determine study eligibility. Studies that met inclusion criteria underwent a validity assessment and data extraction. The reason for rejecting studies were recorded for each study.

Data were extracted and recorded using extraction forms.^25^ The following variables were assessed: 1) type of study, 2) characteristics of the participants, including definition of malocclusion, 3) follow-up duration, 4) characteristics of the intervention, 5) sample size, 6) outcome measures, 7) method of randomization, 8) blindness of examiners, and 9) source of funding and conflicts of interest.

### Risk of bias

Risk of bias of the included studies was evaluated according to the Cochrane Collaboration’s Tool for Assessing Risk of Bias. Briefly, the randomization and allocation methods (selection bias); completeness of the follow-up period/incomplete outcome data (attrition bias); blinding of patients (performance bias) and examiners (detection bias); selective reporting (reporting bias); and other forms of bias were classified as adequate (+), inadequate (-), or unclear (?). Based on these domains, overall risk of bias was categorized as follows: (1) low risk of bias if all criteria were met (adequate methods of randomization and allocation concealment, a “yes” answer to questions about completeness of follow-up and blinding, and a “no” answer to selective reporting and other sources of bias); (2) unclear risk of bias if one or more criteria were partially met; or (3) high risk of bias if one or more criteria were not met.

The methodological quality of the observational studies was assessed using the Newcastle-Ottawa scale (NOS).

## RESULTS

### Articles

**Figure 1:**
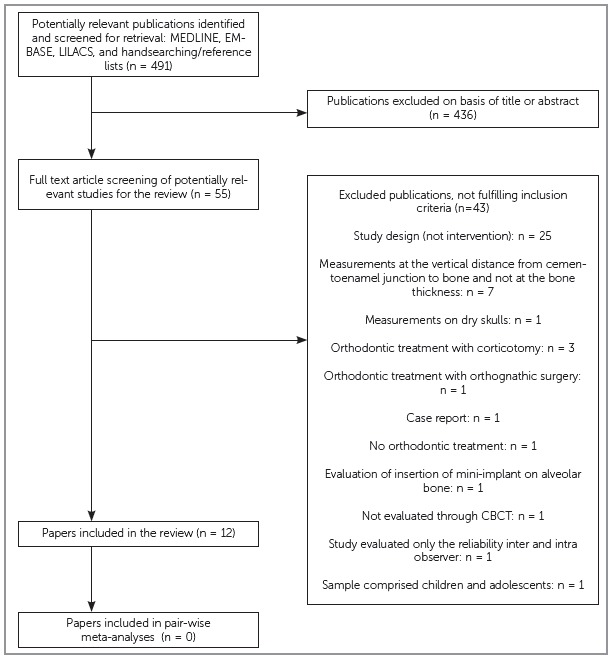
PRISMA diagram of article retrieval.

Initially, 491 references were electronically selected. No additional article was manually identified. After title and abstract evaluation, 436 papers were excluded. The full texts of the remaining 55 publications were considered for detailed reading. Of these publications, 12 were considered eligible for inclusion (Fig 1). 

### Included studies

The characteristics of the included studies are shown in Table 1. From the 12 selected studies, two randomized controlled clinical trials,^26^ ^,^ ^27^ three controlled clinical trials,^28^ ^-^ ^30^ and seven case series^31^ ^-^ ^37^ were found. The studies were conducted in South Korea,^31^ Brazil,^26^ ^,^ ^29^ ^,^ ^37^ Denmark,^27^ Italy,^28^ India,^32^ Turkey,^33^ China,^30^ Thailand,^34^ ^,^ ^36^ and United States.^35^ Research foundations or universities supported three studies.^30^ ^,^ ^34^ ^,^ ^36^ A software company supported one study.^27^ None of the studies reported the follow-up period. A total of 291 orthodontic patients were included in the studies. In the selected articles various types of orthodontic treatment were evaluated and compared. Straight wire appliances with self-ligated and conventional brackets were compared.^26^ Among the different types of self-ligated brackets, passive and active appliances were compared.^27^ Some articles reported orthodontic treatment with Edgewise appliances.^28^ ^,^ ^29^ ^,^ ^32^ ^,^ ^37^ Among the different types of orthodontic treatment, the treatment featuring extraction of the upper first premolars were compared with treatment without extractions.^28^ ^,^ ^29^ Seven studies reported the periodontal status of the patients prior to orthodontic treatment and excluded patients with periodontal disease.^27^ ^,^ ^31^ ^-^ ^36^

**Table 1:** Characteristics of the studies.

Study	Country	Study design	Follow-up		Sample size (baseline)	CBCT Specifications	Source of funding
Ahn et al.^31^, 2013	South Korea	Case series	Not reported		n = 37 female Age range: 26.6 ± 8.5 years	Implagraphy, 12x9-cm field of view, 90-kVp, 4.0-mA tube current, 0.2-mm voxel size and 24-second scan time	No
Almeida et al.^26^, 2015	Brazil	RCT	Not reported		n= 25 (sex distribution not mentioned) Age mean (years): 18.58 ± 5.43 (test); 21.61 ± 6.69 (control)	i-CAT Imaging Sciences International, 22x16-cm field of view, 120 kVp, 36 mA, 0.4-mm voxel size and 40-second scan time	No
Cattaneo et al.^27^, 2011	Denmark	Parallel RCT	Not reported		n= 64 (sex distribution not mentioned) Age mean (years): 16.0 ± 5.7 (test); 15.0 ± 3.3 (control)	NewTom 3G, 12 in field of view, 0.36-mm voxel size	CMF Software (M.E. Muller Institute for Surgical Technology and Biomechanics, University of Bern, Switzerland, developed under the funding of the CO-ME Network)
Lombardo et al.^28^, 2013	Italy	Controlled clinical trial	Not reported		n= 22 (10 male and 12 female) Age mean (years): 11.9 (test); 10.11 (control)	NewTom 3G, 12 in field of view, 110- kV, 2.00 mA, 5.4 second exposure time	No
Nayak-Krishna et al.^32^, 2013	India	Case series	Not reported		n= 10 (sex distribution not mentioned) Age range: 15 ± 3 years	GE medical systems, 120 kV, 160 mva	No
Oliveira et al.^37^, 2016	Brazil	Case series	Not reported		n= 11 (5 male and 6 female) Age range: 18 to 26 years old	i-CAT Imaging Sciences International, 13x17-cm field of view, 120 kVp, 5 mA, 0.4-mm voxel size and 20-second scan time	No
Picanço et al.^29^, 2013	Brazil	Controlled clinical trial	Not reported		n= 12 (10 male and 2 female) Age mean: 15.83 ± 4.87 years (test); 18.26 ± 6.42 years (control)	Not reported	No
Sarikaya et al.^33^, 2002	Turkey	Case series	Not reported		n = 19 (sex distribution not mentioned) Age mean: 14.1 ± 2.3 years	Tomoscan SR7000, 120 kV, 175 mA and 1.5-mm slice thickness	No
Sun et al.^30^, 2015	China	Controlled clinical trial	Not reported		n= 42 (sex distribution not mentioned) Age mean: not mentioned	Galileo, 150-mmx150-mm field of view, 85 kV, 21 mA, 20 second exposure time	This work was supported by the School Funds of Jinling Hospital, School of Medicine, Nanjing University (No. 2013079). Open Science Foundation for National Key Laboratory of Military Stomatology (No. 2014KC02), and China Postdoctoral Science Foundation (No. 2015M572814)
Thongudomporn et al.^34^, 2015	Thailand	Case series	Not reported		n = 15 (4 male and 11 female) Age mean: 9.9 ± 1.0 years	Veraviewepocs J Morita MPG, 80 kV, 5 mA, 7.5 second exposure time, 0.125 mm voxel resolution, 80 x 40 mm field of view	Grant support from Graduate School and the Faculty of Dentistry, Prince of Songkla University
Uribe et al.^35^, 2013	USA	Case series	Not reported		n = 11 (7 male and 4 Female) Age range: 16.45 ± 5.76 years	i-CAT Classic scanner, 20-second scan time with a 16-cm x 13-cm field of view at a resolution of 0.3-mm voxels, 120 kVp, 3-8 mA	No
Yodthong et al.^36^, 2013	Thailand	Case series	Not reported		n = 23 (2 Male and 21 Female) Age range: 20.4 ± 2.7 years	Veraviewepocs J Morita MPG, 80 kV, 5 mA, 7.5 second exposure time, 0.125 mm voxel resolution, 80 x 40 mm field of view	Graduate School, Faculty of Dentistry, Prince of Songkla University, for grant support

### Quality assessment

**Figure 2:**
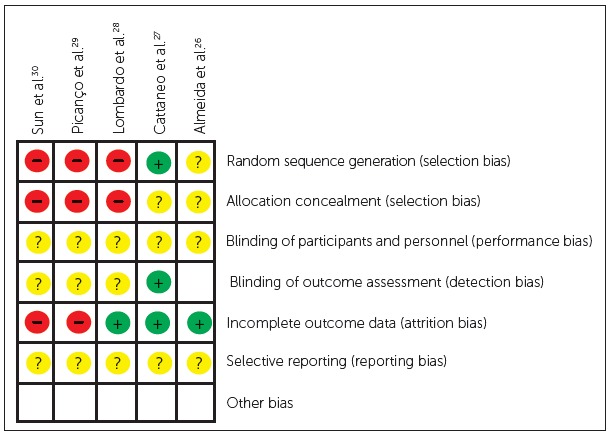
Risk of bias summary.

Among the 5 clinical trials, only one reported an adequate method of randomization.^27^ None of the trials reported an adequate method of allocation concealment. Only one article described blinding of examiners on treatment procedures.^27^ No study mentioned blinding of participants. The number of patients at baseline and final examination was described in three articles.^26^ ^-^ ^28^ Therefore, based on the criteria established by the present review, three studies^28^ ^-^ ^30^ were considered to present a high risk of bias and two studies^26^ ^,^ ^27^ were considered to have unclear risk of bias (Fig 2). Furthermore, only one trial^26^ reported sample size calculation.

In the 7 case series, the Newcastle-Ottawa scale (NOS) was used to verify methodological quality (Tab 2). NOS scale was adapted for the purpose of this review, and each included study received a maximum of 14 points. Studies with 9-14 points were considered as presenting high methodological quality; 6-8 points studies, as medium quality; and those with <6 points, as presenting low methodological quality. Of the seven included studies, two received a 7-point score,^31^ ^,^ ^32^ one a 3-point score^33^ and four a 6-point score^34^ ^-^ ^37^(Table 2). Thus, 6 studies were considered as medium methodological quality and 1 as low methodological quality. Two studies reported sample size calculation,^34^ ^,^ ^37^ and no study gave information about training of assessors, comparability of groups on the basis of the design, assessment of clinical conditions and adequacy of follow-up patients. In all of the included studies, ascertainment of the bone before orthodontic treatment and validity of statistical analysis were considered adequately addressed.

**Table 2:** Methodological quality evaluation of included studies using Newcastle-Ottawa scale.

Study		Ahn et al.^31^	Nayak-Krishna et al.^32^	Oliveira et al.^37^	Sarikaya et al.^33^	Thongudomporn et al.^34^	Uribe et al.^35^	Yodthong et al.^36^
Selection	Sample size calculation	0	0	*	0	*	0	0
	Representativeness of orthodontic patients	*	*	0	*	*	0	0
	Selection of the orthodontic control group	0	0	0	0	0	0	0
	Ascertainment of the bone before orthodontic treatment	*	*	*	*	*	*	*
	Outcome of interest not present at the start	0	*	0	0	0	0	*
	Training of assessors	0	0	0	0	0	0	0
	Description of inclusion/exclusion criteria	*	*	*	0	*	*	*
Comparability	Comparability of groups on the basis of the design	0	0	0	0	0	0	0
	Management of confounders	*	*	*	0	0	*	*
Outcome	Assessment of clinical conditions	0	0	0	0	0	0	0
	Definitions and assessment of bone resorption clearly reported	*	*	*	0	*	*	*
	Adequacy of follow-up of patients	0	0	0	0	0	0	0
Statistics	Validity of statistical analysis	*	*	*	*	*	*	*
	Unit of analysis reported	*	0	0	0	0	*	0
Total	(14/14)	7/14	7/14	6/14	3/14	6/14	6/14	6/14

### Effects of interventions

#### Different types of treatment

Twelve studies assessed changes in bone thickness as a result of the orthodontic movement (Table 3). Five trials showed a significant reduction in bone thickness associated with retraction of anterior teeth with maximum anchorage. Among them, one study was performed with self-ligated appliance,^31^ three studies used edgewise appliance^28^ ^,^ ^29^ ^,^ ^32^ and one study used 0.018 x 0.025-in Roth appliance.^33^ Four trials that did not use treatment with premolar extractions also showed reduction on bone thickness. Among them, one study compared self-ligated and conventional straight-wire appliances^26^ and did not find differences between the two techniques regarding buccal bone plate changes; one study compared two different types of self-ligated straight wire appliances - passive and active - and also did not find differences between groups;^27^ and two studies used conventional Straight-wire appliances.^30^ ^,^ ^34^ Two studies didn’t report the technique but described reduction in alveolar bone.^35^ ^,^ ^36^ One study reported extraction of maxillary first premolars and retraction of maxillary incisors, and showed no statistically significant differences of alveolar thickness before and after treatment.^37^

**Table 3:** Individual studies outcomes.

Articles	Teeth	Groups	Views	Results*
Ahn et al.^31^, 2013	Maxillary central incisors Maxillary lateral incisors Maxillary canines	Test group (Class I dentoalveolar protrusion treated with extraction of the four first premolars and sliding mechanics and straight-wire appliance)	Buccal side Palatal side	CENTRAL INCISORS Buccal: Cervical: -0.16 ± 1.89 mm / Middle: 0.65 ± 1.47 mm / Apical: -0.19 ± 2.42 mm Palatal: Cervical: -1.82 ± 1.18 mm / Middle: -4.32 ± 3.09 mm / Apical: -6.66 ± 6.62 mm LATERAL INCISORS Buccal: Cervical: 0.05 ± 0.98 mm / Middle: 0.84 ± 1.49 mm / Apical: 0.09 ± 1.62 mm Palatal: Cervical: -1.40 ± 0.94 mm / Middle: -3.38 ± 3.30 mm / Apical: -6.17 ± 6.27 mm CANINES Buccal: Cervical: 0.64 ± 2.67 mm / Middle: 0.40 ± 1.82 mm / Apical: -0.10 ± 3.73 mm Palatal: Cervical: -1.57 ± 2.11 mm / Middle: -2.29 ± 6.44 mm / Apical: -8.25 ± 13.51 mm
Almeida et al.^26^, 2015	Mandibular first premolar Mandibular second premolar Mandibular first molar	Test group (Class I malocclusion treated with self-ligated brackets) Control group (Class I malocclusion treated with conventional brackets)	Buccal side at apical height	Test Group First premolar: -0.77 ± 1.46 mm / Second premolar: -0.86 ± 0.72 mm First molar: -0.43 ± 0.76 mm Control Group First premolar: -1.20 ± 1.64 mm / Second premolar: -0.98 ± 1.78 mm / First molar: -0.55 ± 0.91 mm
Cattaneo et al.^27^, 2011	First premolar	Test group (Damon passive self-ligated brackets) Control group (In-Ovation active self-ligated brackets)	Buccal cortical bone plate	Damon T_1_ - T_0_(n=32) Upper premolar: -0.1 ± 3.27 mm In-Ovation T_1_-T_0_(n=32) Upper premolar: -2.25 ± 3.20 mm
Lombardo et al.^28^, 2013	Mandibular first premolar Maxillary second premolar	Test group (Class II division 1 malocclusion with extraction of the upper first premolars and lower second premolars treated with Tweed technique) Control group (orthodontic treatment without extraction with Tweed technique)	Buccolingual thickness (BT)	Test group First premolar BT changes: 3.19 mm Second premolar BT changes: 1.71 mm Control group First premolar BT changes: 0.98 mm Second premolar BT changes: 0.67 mm
Nayak-Krishna et al.^32^, 2013	Maxillary central incisors Maxillary lateral incisors Mandibular central incisors Mandibular lateral incisors	Test group (patients with bimaxillary dentoalveolar protrusion treated with extraction of first premolars and edgewise technique)	3 mm 6 mm 9 mm Buccal bone thickness (BBT) Lingual bone thickness (LBT)	Maxillary central incisors BBT: 3 mm: 0.30 ± 0.5 mm / 6 mm: 0.20 ± 0.44 mm / 9 mm: 0.10 ± 0.76 mm Maxillary central incisors LBT: 3 mm: 0.40 ± 0.77 mm / 6 mm: 0.50 ± 0.54 mm / 9 mm: -0.40 ± 0.54 mm Maxillary lateral incisors BBT: 3 mm: 0.40 ± 0.5 mm / 6 mm: 0.30 ± 0.5 mm / 9 mm: 0.50 ± 0.77 mm Maxillary lateral incisors LBT: 3 mm: 0.10 ± 0.61 mm / 6 mm: 0.40 ± 0.5 mm / 9 mm: 0.40 ± 0.76 mm Mandibular lateral incisors BBT: 3 mm: 0.10 ± 0.42 mm / 6 mm: 0.30 ± 0.45 mm / 9 mm: 0.30 ± 0.72 mm Mandibular lateral incisors LBT: 3 mm: 0.40 ± 0.97 mm / 6 mm: -0.30 ± 0.80 mm / 9 mm: -0.50 ± 0.51 mm Mandibular central incisors BBT: 3 mm: 0.30 ± 0.46 mm / 6 mm: 0.40 ± 0.51 mm / 9 mm: 0.30 ± 0.74 mm Mandibular central incisors LBT: 3 mm: 0.40 ± 0.51 mm / 6 mm: -0.30 ± 0.46 mm / 9 mm: -0.60 ± 0.51 mm
Articles	Teeth	Groups	Views	Results*
Oliveira et al.^37^, 2016	Maxillary central and lateral incisors	Test group (Class II division 1 and Class I malocclusion treated with extraction of first premolars and edgewise technique)	Alveolar bone width measurements at 2, 4, 6, 8, 10, 12 and 14 mm apical to the alveolar crest	Bone width at 2 mm: RD: -1.11 ± 0.61 / RM: -0.9 ± 0.13 mm / ML: 0.22 ± 1.11 mm / LM: -0.8 ± 0.13 mm / LD: -1.12 ± 0.63 mm Bone width at 4 mm: RD: -0.99 ± 0.67 mm / RM: -0.77 ± 0.61 mm / ML: 0.49 ± 1.3 mm / LM: -0.81 ± 0.36 mm / LD: -0.61 ± 0.18 mm Bone width at 6 mm: RD: -0.06 ± 0.86 mm / RM: 0 ± 0.32 mm / ML: 0.33 ± 0.29 mm LM: -0.63 ± 0.06 mm / LD: 0.01 ± 0.27 mm Bone width at 8 mm: RD: -0.06 ± 0.49 mm / RM: 0.18 ± 0.65 mm / ML: 0.22 ± 0.51 mm LM: -0.23 ± 0.16 mm / LD: 0.02 ± 0.03 mm Bone width at 10 mm: RD: 0.47 ± 0.65 mm / RM: 0.39 ± 0.43 mm / ML: 0.22 ± 0.41 mm LM: 0.38 ± 0.11 mm / LD: 0.42 ± 0.41 mm Bone width at 12 mm: RD: 0.04 ± 0.13 mm / RM: 0.56 ± 0.96 mm / ML: 0.46 mm ± 0.91 LM: 0.39 ± 0.48 mm / LD: 0.08 ± 0.24 mm Bone width at 14 mm: RD: -0.58 ± 4.38 mm / RM: -1.19 ± 4.66 mm / ML: -1.33 ± 2.68 mm LM: -1.11 ± 4.62 mm / LD: 0.72 ± 0.08 mm
Picanço et al.^29^, 2013	Maxillary central incisors	Test group (Class II malocclusion treated with upper premolar extraction) Control group (Class I and Class II malocclusion treated without extraction)	Buccal and palatal sides at 3 mm, 6 mm and 9 mm from cemento-enamel junction UL: Buccal alveolar bone UP: Palatal alveolar bone	Group 1 (n=6): UL cerv.: 0.63 ± 0.49 mm / UP cerv.: -1.39 ± 0.51 mm / UL midpoint: 1.15 ± 1.27 mm / UP midpoint: -1.62 ± 0.86 mm / UL apical: 1.95 ± 2.98 mm / UP apical: -1.54 ± 2.57 mm Group 2 (n=6): UL cerv.: -0.06 ± 0.47 mm / UP cerv.: -0.66 ± 0.90 mm / UL midpoint: 0.16 ± 0.86 mm / UP midpoint: -0.80 ± 0.76 mm / UL apical: 0.00 ± 0.74 / UP apical: -0.56 ± 1.51 mm
Sarikaya et al.^33^, 2002	Maxillary central incisors Maxillary lateral incisors Mandibular central incisors Mandibular lateral incisors	Test group (patients with dentoalveolar bimaxillary protrusion treated with extractions of the 4 first premolars and straight-wire appliances)	Buccal and palatal sides at 3 mm, 6 mm and 9 mm from cemento-enamel junction	LABIAL Maxillary central incisors 3 mm: -0.24 ± 0.57 mm / 6 mm: -0.03 ± 0.64 mm / 9 mm: 0.06 ± 0.89 mm Maxillary lateral incisors 3 mm: -0.26 ± 0.53 mm / 6 mm: 0.20 ± 0.50 mm / 9 mm: 0.19 ± 0.64 mm LINGUAL Maxillary central incisors 3 mm: -0.93 ± 0.69 mm / 6 mm: -1.12 ± 0.06 mm / 9 mm: -0.58 ± 1.33 mm Maxillary lateral incisors 3 mm: -1.11 ± 0.58 mm / 6 mm: -0.97 ± 0.91 mm / 9 mm: -0.67 ± 1.48 mm LABIAL Mandibular central incisors: 3 mm: -0.28 ± 0.44 mm / 6 mm: -0.05 ± 0.68 mm / 9 mm: -0.28 ± 1.04 mm Mandibular lateral incisors: 3 mm: -0.38 ± 0.28 mm / 6 mm: -0.22 ± 0.52 mm / 9 mm: -0.30 ± 0.89 mm LINGUAL Mandibular central incisors: 3 mm: -0.87 ± 0.41 mm / 6 mm: -0.52 ± 0.75 mm / 9 mm: -0.46 ± 0.13 mm Mandibular lateral incisors: 3 mm: -0.66 ± 0.5 mm / 6 mm: -0.49 ± 0.78 mm / 9 mm: -0.22 ± 0.97 mm
Sun et al.^30^, 2015	Mandibular incisors	Test group (patients with Class III malocclusion treated with straight-wire appliance) Control group (patients with normal occlusion)	Labial alveolar bone thickness at apical level Lingual alveolar bone thickness at apical level	Labial alveolar bone thickness: 1.71 ± 0.43 mm Lingual alveolar bone thickness: -2.07 ± 0.51 mm
Articles	Teeth	Groups	Views	Results*
Thongudomporn et al.^34^, 2015	Maxillary incisors	Test group (patients with mild skeletal Class III and straight-wire appliances)	Buccal and palatal sides at 3 mm, 6 mm and 9 mm from cemento-enamel junction	Labial alveolar thickness: 3 mm: -0.12 ± 0.18 mm / 6 mm: -0.18 ± 0.31 mm / 9 mm: -0.01 ± 0.63 mm Palatal alveolar thickness: 3 mm: -0.13 ± 0.24 mm / 6 mm: -0.34 ± 0.30 mm / 9 mm: -0.59 ± 0.48 mm
Uribe et al.^35^, 2013	Maxillary central incisors Maxillary canines	Test group (patients with unilaterally or bilaterally congenitally missing maxillary lateral incisors)	Alveolar bone width measurements at 2, 4, 6, 8, and 10 mm apical to the alveolar crest	Central incisor: 2 mm: -0.45 ± 0.55 mm / 4 mm: -0.55 ± 0.76 mm / 6 mm: -0.88 ± 1.13 mm 8 mm: -1.35 ± 1.22 mm / 10 mm: -1.29 ± 1.64 mm Canine: 2 mm: -0.80 ± 1.17 mm / 4 mm: -0.67 ± 1.12 mm / 6 mm: -0.29 ± 1.27 mm 8 mm: -0.01 ± 1.43 mm / 10 mm: -0.07 ± 1.59 mm
Yodthong et al.^36^, 2013	Maxillary incisors	Test group (patients receiving orthodontic treatment with upper incisors bound to retraction with a space >4 mm between lateral incisors and canines	Labial alveolar thickness and palatal alveolar thickness at crestal, midroot and apical levels.	Labial thickness at crestal level: -0.4 ± 0.3 mm Palatal thickness at crestal level: 0.20 ± 0.36 mm Labial thickness at midroot level: -0.2 ± 0.3 mm Palatal thickness at midroot level: -0.1 ± 0.60 mm Labial thickness at apical level: 0 ± 0.3 mm Palatal thickness at apical level: -0.6 ± 1.41 mm

* Difference between pretreatment and post treatment values as regards alveolar bone thickness. Negative values indicate a reduction in bone width; ML: midsagittal plane; RM 5mm apart from ML to the right; RD 10 mm apart from ML to the right; LM 5mm apart from ML to the left; LD 10mm apart from ML to the left.

### Different tooth evaluated

Nine studies evaluated central and lateral incisors.^29^ ^-^ ^37^ Among them, eight studies evaluated maxillary incisors and three studies evaluated mandibular incisors.^30^ ^,^ ^32^ ^,^ ^33^ One study evaluated mandibular premolars,^26^ two studies evaluated maxillary premolars,^27^ ^,^ ^28^ two studies evaluated maxillary canines^31^ ^,^ ^35^ and one study evaluated mandibular molars.^26^

### Distance from CEJ

Seven studies evaluated bone thickness changes at different distances from CEJ. Three studies evaluated bone changes at 3, 6 and 9mm from CEJ.^29^ ^,^ ^32^ ^,^ ^33^ Two studies reported changes in alveolar thickness at crestal, midroot and apical areas,^34^ ^,^ ^36^ one study reported evaluation at 2, 4, 6, 8 and 10 mm from CEJ^35^ and one study reported evaluation at 2, 4, 6, 8, 10, 12 and 14 mm from CEJ.^37^

Regarding studies that evaluated the bone thickness on incisors at different levels from CEJ, only one study found a significant bone loss only at the cervical,^33^ while other studies found an increase in this region^29^ ^,^ ^36^. Some studies found bone loss at multiple sites.^31^ ^,^ ^32^ ^,^ ^34^ ^,^ ^37^ In addition, some studies reported bone loss at the buccal side of incisors,^32^ ^,^ ^33^ while others reported an increase in buccal bone.^29^ ^,^ ^36^

Most of the studies showed that orthodontic treatment produces a reduction in bone thickness.^26^ ^-^ ^28^ ^,^ ^31^ ^-^ ^35^ The reduction in bone thickness was more pronounced at the palatal side, especially at incisors. 

## DISCUSSION

In spite of many studies investigating the association of bone resorptions and orthodontic treatment, this is the first systematic review to assess the effects of orthodontic treatment on bone remodeling. Although the 12 selected studies are very heterogeneous, it can be observed that most of the studies showed that orthodontic treatment produces a reduction in bone thickness. No meta-analysis could be performed because the studies included different orthodontic techniques, evaluated distinct teeth with diverse forces. Furthermore, variable treatment times were found.

Studies that performed measurements of bone remodeling without the usage of CBCT were excluded. CBCT enables examination of alveolar bone morphology with quality, since three-dimensional images are not subject to distortion or superimposition.^23^ ^,^ ^24^

Regarding the specification of the force used in tooth movement, the only authors who reported the measurement of force used in orthodontic treatment were: Nayak-Krishna et al,^32^ who reported light continuous forces of 100g for retraction of anterior teeth; Ahn et al,^31^ who reported 200g of force on elastic chains also to retract anterior teeth; Thongudomporn et al,^34^ who placed 89.6g on upper incisors for buccal tipping movements; and Oliveira et al,^37^ who activated the incisors retraction with a force of 150g per side. The individual analysis of the selected studies does not imply that a specific type of force causes more alveolar bone loss than others. 

The articles were heterogeneous regarding the type of orthodontic movement performed. As to the treatment plan, some articles reported only alignment and leveling movements^26^ ^,^ ^27^ ^,^ ^30^ and other studies performed premolars extractions with retraction of the anterior teeth.^28^ ^-^ ^33^ ^,^ ^36^ ^,^ ^37^ Thongudomporn et al^34^ treated patients with anterior crossbite through buccal tipping and extrusion of upper incisors using advancing loops and Class III elastics.

The heterogeneity of the studies also comprised the type of orthodontic bracket and technique; thus few studies used Straight-wire technique,^26^ ^,^ ^27^ ^,^ ^30^ ^,^ ^31^ ^,^ ^33^ ^,^ ^34^ while others performed Edgewise technique^28^ ^,^ ^29^ ^,^ ^32^ ^,^ ^37^. Almeida et al.^26^ compared self-ligating with conventional brackets, while Cattaneo et al.^27^ compared different types of self-ligating brackets. The heterogeneity and the quality of the included studies are the limitations of this study.

Regarding the regions where bone changes were measured, the following sites were analyzed: alveolar bone area at cervical, middle and apical levels,^29^ ^,^ ^31^ ^-^ ^34^ most external proeminence of the buccal bone in the most apical portion of the root,^26^ alveolar bone width measurements at 2, 4, 6, 8, 10, 12 and 14 mm apical to the alveolar crest^35^ ^,^ ^37^ and labial and palatal bone thickness at the crestal level, midroot level and apical level.^36^

The absence or insufficiency of alveolar bone thickness is a complicating factor for orthodontic treatment. The occurrence of dehiscences and fenestrations during orthodontic treatment depends on factors such as: direction of movement, frequency and magnitude of orthodontic forces, volume and anatomical integrity of periodontal tissues.^4^

As regards the implications for dental practice, we consider that these findings are relevant and have to be considered not only in diagnosis but also in the planning of tooth movement, in order to prevent the occurrence of dehiscence and fenestration in the alveolar bone. Additionally, it is interesting to notice that the majority of the studies observed a higher percentage of bone remodeling at the palatal side. Probably, the reason for this greater effect at the palatal side is a result of the type of orthodontic movement (retraction of the incisors).^33^ The loss of alveolar bone at the palatal side doesn’t have an impact on esthetics, but it has to be considered on the orthodontic treatment, since if the patient does not undergo a rigid periodontal follow-up it can result in severe and definitive loss of periodontal support. We emphasize the need for periodontal diagnosis; strict dental biofilm control and regular maintenance visits for patients undergoing orthodontic treatment. 

## CONCLUSION

On patients undergoing different orthodontic treatment techniques, there was a significant bone thickness reduction, mainly on the palatal side. However, the results should be interpreted with caution, because of the heterogeneity of the included studies.
